# Condom application skills and self-efficacy in youth: A systematic review and meta-analysis

**DOI:** 10.1371/journal.pone.0249753

**Published:** 2021-04-08

**Authors:** John L. Ferrand, Aaron J. Blashill, Heather L. Corliss, Eric R. Walsh-Buhi

**Affiliations:** 1 Department of Applied Health Science, School of Public Health, Indiana University Bloomington, Bloomington, IN, United States of America; 2 Department of Psychology, San Diego State University, San Diego, CA, United States of America; 3 San Diego State University/University of California Joint Doctoral Program in Clinical Psychology, San Diego, San Diego, CA, United States of America; 4 School of Public Health, San Diego State University, San Diego, CA, United States of America; George Washington University, UNITED STATES

## Abstract

Globally, and in the United States (U.S.) specifically, rates of reported sexually transmitted infections (STIs) have been steadily increasing and are especially high among youth aged 13–25 years. Using condoms correctly and consistently is an effective STI prevention measure for sexually active youth, yet public health endeavors tend to focus only on condom use consistency. Directly measuring condom application is challenging and expensive. Alternative tools evaluate this behaviour, but little evidence exists on the appropriateness of these instruments in measuring application skills. This systematic review and meta-analysis examined the association between condom application skills and self-efficacy. We conducted a search of several databases as well as unpublished works. Studies were included if they were in English, examined youth aged 13–25 years, and were available between 1992 and 2019. The authors screened 630 titles and abstracts for initial inclusion criteria. A full-text review of 30 studies was conducted. The authors included 19 studies in the systematic review and 5 studies were included in the meta-analysis. Both a fixed- and random-effects model (Q = .2321, I^2^ = 0%) yielded a medium-sized statistically non-significant association (*r* = 0.217) between skills and self-efficacy. Despite the small sample size, findings suggest that skills and self-efficacy may not be as interchangeable as previously assumed when assessing condom application. Implications for future research are discussed.

## Introduction

Globally, the occurrence of sexually transmitted infections (STIs) are high, with an estimated 127.2 million, 86.9 million, and 6.3 million new incidents of chlamydia, gonorrhoea, and syphilis, respectively, in 2016 [1]. The U.S. has a similarly high rate of STI occurrence. In fact, STIs occurred at a higher rate in the year 2017 than they did at any point in the past two decades, with reported rates of 9.5, 528.8, and 171.9 per 100,000 people of all ages for syphilis (primary and secondary), chlamydia, and gonorrhoea, respectively [2]. Additionally, surveillance data indicate that the rate of occurrence of chlamydia and gonorrhea are highest for those aged 20–24 years, with 2644.0 and 607.5 per 100,000 people, respectively [3]. Similarly, rates of human immunodeficiency virus (HIV) are highest among individuals aged 20–29 years (34.8 per 100,000 people; [3]. Trend data from 2017 paint a similarly bleak portrait of STI occurrences in the U.S., with a 67% and a 76% increase in gonorrhea and syphilis diagnoses, respectively, since 2013 [2].

### Significance of condom application skills

Although a minority of studies have reported mixed findings [4–6], overall, correct and consistent condom use remains an effective HIV and STI prevention measure [7, 8]. Yet, efforts to improve sexual health outcomes tend to focus on consistent use over correct use [9], despite the many ways that incorrect use can impede condom effectiveness [10]. Several systematic reviews indicated that sexual health promotion programs frequently focus on increasing condom use consistency, and some of these programs were successful at increasing the frequency in which condoms are reportedly utilized [11, 12]. However, there is evidence that youth aged 15–23 years have incorrect information regarding condom use that could lead to subsequent incorrect application (e.g., not leaving space at the tip, using oil-based lubricants; [13]). Incorrect condom application includes behaviors such as opening the package incorrectly, putting the condom on the penis backwards, removing the condom in the middle of sexual activity, and using a condom more than one time [9, 14]. A study of sexually active women reported that those between the ages of 21–25 years were at a higher risk of incorrectly applying a condom than younger women in the study [15]. More importantly, almost one-third (32%) of women in the study who reported consistent condom use committed application errors (e.g., re-using a condom or genital contact before or after condom use) compared to over two-fifths (43%) of women who reported inconsistent condom use. These findings are similar to that of a study of men enrolled in college where consistent and inconsistent condom users were equally likely to report condom application errors [16]. This suggests that frequent condom use without adequate application skills training may leave individuals with a trial and error approach to correct condom application. For the purposes of this study, condom application must include the act of applying the condom to, and removing the condom from, a penis either as reported by a participant or as observed by a research investigator. Studies that included other aspects of condom use, such as purchasing condoms or negotiating their use, but did not include the actual application and removal of the condom were excluded.

The significance of correct condom application has been documented in studies highlighting the association between incorrect application and subsequent condom failure [9, 14, 16–18]. Several studies conducted in STI treatment clinics attempted to further validate the association between self-reported or observed incorrect condom application and STI transmission by assessing for the presence of biological indicators of STI transmission such as prostate specific antigen detection in vaginal swabs following reported condom use. Using this method, Crosby et al. [19] found that correct condom application combined with consistent condom use significantly reduced one’s odds of STI transmission whereas Duerr et al. [17] found that, while not every condom error resulted in condom failure, several were associated with semen exposure (e.g., touching tip of penis with hand before applying condom). Despite the demonstrated importance of correct condom application in those studies, few investigators assess application skills, choosing instead to measure a different dimension of condom use, such as self-efficacy or attitudes towards condom use, which have not been comprehensively evaluated as appropriate approximations of one’s condom application skills. These dimensions are frequently used as approximations for one’s condom application skills possibly due to their coexistence in well-known behavioural theories such as the Social Cognitive Theory and the Information-Motivation-Behavioural Skills Model [20–22]. In fact, in a review of condom use measurements, self-efficacy was cited as the most frequently assessed construct when measuring condom use correctness [23]. Given these findings, the next logical step in evaluating sexual health programs and interventions should be to further assess how accurately participants use condoms, but at the time of this study, no actual efforts have been made to do so.

### Evaluation of condom application skills

As noted above, correct condom application is effective in preventing negative sexual health outcomes [10, 24, 25], yet it remains a complex and multifaceted behavior that is challenging to measure [23, 26, 27]. Instruments have been developed to measure condom application skills through knowledge tests of condom application steps [28] or by observing participants while they apply a condom to a penile model and rating their performance [29–32]. Unfortunately, studies utilizing direct observation methodologies are scarce. The scarcity of direct observation instruments and approaches is not surprising considering the difficulty in conducting a skills assessment of condom application. As mentioned, different instruments tend to assess different skills related to correct condom application with some instruments overlapping in items and some assessing entirely different dimensions of condom application. For instance, the Condom Use Directly Observable Skill (CUDOS) measure [32], the Measure of Observed Condom Use Skills (MOCUS) scale [31], and the Male Condom Use Skills (MCUS) measure [29] contain similar items for assessing the actual demonstrated application of the condom and the removal of the condom, but the MCUS evaluates the selection of appropriate lubricants and latex condoms whereas the CUDOS only evaluates the selection of a latex condom and the MOCUS does not evaluate either of these dimensions. This makes knowing what skills to measure difficult. In addition to the varying skills a scale or survey purports to assess, conducting the skills assessment, whether it be direct observation or self-report, has often been met with resistance from multiple entities (e.g., legislation banning condom application skills trainings in schools and cultural antipathy towards participating in such an assessment), which reduces the sample of participants [33]. These barriers to effectively measuring condom application skills led to the adoption of alternate methods to quantify the behavior, many of which have not been evaluated as appropriate approximations of condom application skills.

Some research and behavioral interventions for youth have incorporated condom use skills training along with assessments of knowledge and attitudes towards condoms, but few researchers assess these outcomes using the same instruments, with evidence of reliability and validity, making it difficult to draw comparisons between programs with different outcome measures [34]. While responses to distinct instruments are not impossible to compare, the operationalization of the underlying constructs should be comparable between the instruments. In addition to concerns regarding operationalization of condom application skills, directly assessing condom application skills is challenging for several reasons, such as the time necessary to conduct such an assessment in the midst of other intervention tasks or stakeholder pushback with regards to providing condoms to young people [28, 33, 35–37]. These barriers to directly assessing condom use have stunted progress towards developing and evaluating direct observation instruments. Accordingly, sexual health promotion researchers have utilized alternative methods in approximating this behavior [28, 31], while others have attempted to estimate skills using the concept of self-efficacy applied to condom use [33].

### An alternative to direct observation of condom application skills

#### Self-efficacy

Self-efficacy has been used as a predictor for outcomes, such as academic performance [38–40] and myriad health behaviors such as physical activity and medication adherence [41–45]. Assessing condom use with a self-efficacy scale was more common than with a correct application scale as demonstrated in a systematic review of studies conducted between 1989 and 2003. This review found that of 56 studies examining condom use measurements only 9 studies assessed correct condom use skills, and the majority of those 9 studies used a measure of self-efficacy to assess skills [23]. Another review of studies who reported increases in general condom use indicated that not a single study used a measure of condom application skills [46]. Eventually, scales were developed specifically for assessing self-efficacy for condom use, such as the Condom Use Self-Efficacy Scale (CUSES), which has been used for the past 25 years to assess one’s confidence in applying a condom as well as buying, negotiating their use, and disposing of them [47]. Condom use self-efficacy can also be quite nuanced in its own conceptualization with a number of dimensions included under its auspices such as confidence to talk about condoms with a partner and confidence in using a condom correctly in both non-intoxicated and intoxicated situations [48, 49]. While assessing condom use self-efficacy may circumvent the difficulty in directly assessing condom use skills, little is known about the association between actual condom use skills and condom use self-efficacy among youth, as well as evidence of reliability and validity of data collected via measures to assess such attributes.

### Research questions/hypothesis

In accordance with the Preferred Reporting Items for Systematic Reviews and Meta-Analyses (PRISMA) statement [50], this systematic review and meta-analysis examined the association between measures of condom application skills and measures of condom use self-efficacy to answer the following research question: What is the association between measures of condom application skills and condom use self-efficacy? Due in part to differences in conceptualization and construction between measures of skills and measures of self-efficacy [51], we hypothesized there will be a weak, positive association between these two classifications of measures.

## Materials and methods

### Systematic literature review

#### Preparation

Before conducting the review, we searched for similar reviews in the Cochrane Database of Systematic Reviews and in the journal, *Systematic Reviews*. While no reviews examined both condom use skills and condom use self-efficacy, there were some that examined one of the primary assessment methods, such as condom use self-efficacy. These reviews were mined for keywords that could be used to generate search terms. In addition, we determined that the most similar operationalization of condom use self-efficacy would appear after the development of the CUSES in 1991. Therefore, we decided to begin our search near this time point while also allowing for dissemination of the CUSES. We developed a systematic review protocol and submitted it to an international register of prospective systematic reviews (PROSPERO; CRD42018081960). In preparation for data extraction, we developed an online survey abstraction form, using Qualtrics, that we used to catalog and code study information and characteristics.

#### Search strategy

**Fig 1:**
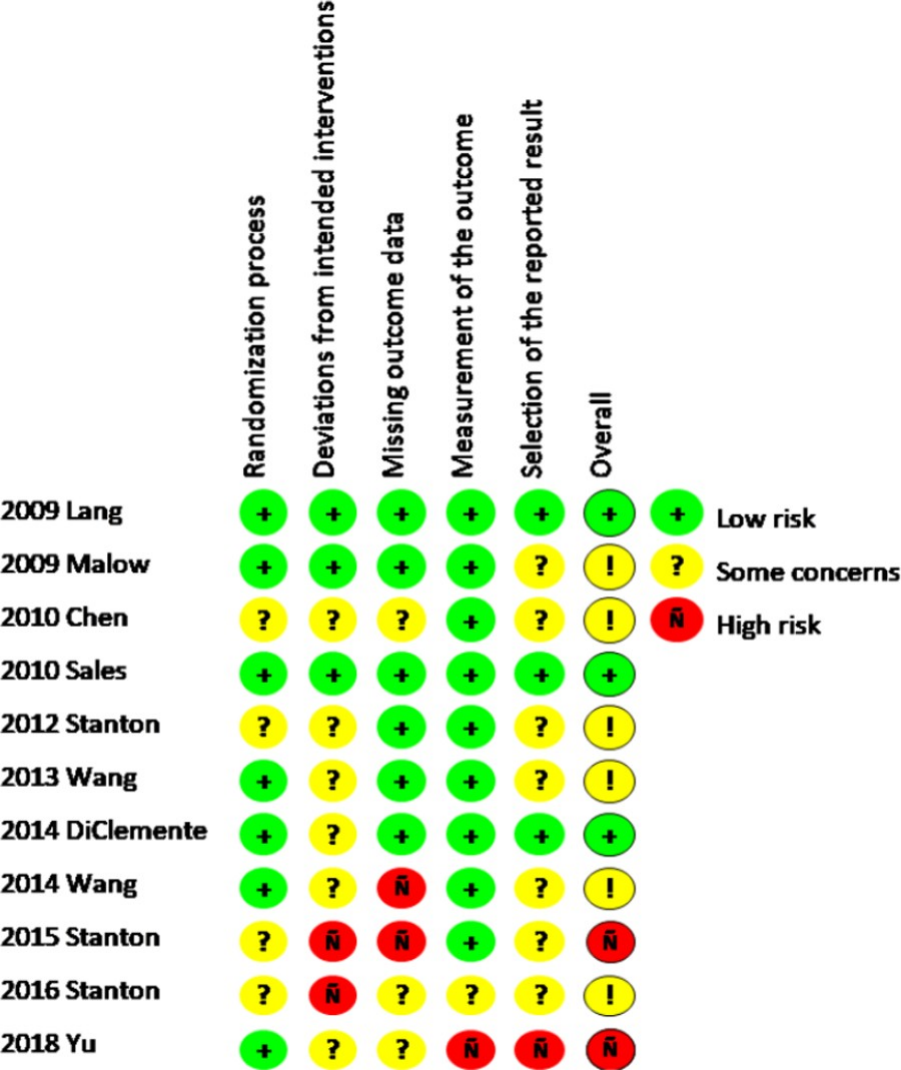
PRISMA flowchart.

Inclusion in this review study was contingent upon studies meeting the following criteria: 1) published in English between January 1992 and December 2019, 2) explicitly mentioned skills and self-efficacy in the article’s methods section, and 3) focused on youth aged 13–25 years of age. While restricting to English language articles may have introduced some additional bias in our pooled sample (as results in English language articles may be more positively skewed [52], the cost associated with translating languages other than English outweighed the risk of bias in this study. The inclusion age range of 13–25 years was chosen to account for some variation in definitions of youth populations [53, 54] which could include teenagers (aged 13–19 years) and young adults (aged 18–25 years). Research suggests that those aged 10–14 years undergo pubertal changes that could result in engaging in sexual activity [55, 56], but trend data from the Youth Risk Behavior Survey [57] suggest that sexual activity is not prevalent in those aged younger than 13 years in the U.S. (3.0% in 2019). With that in mind, we chose 13 years as the lower bound for the age range. We included theses and dissertations if they were indexed within ProQuest Dissertations and Theses. Investigators were contacted regarding unpublished manuscripts, but none were included in this review. We excluded articles if they were not quantitative (e.g., qualitative studies, reviews, editorials). Database searches were conducted through PsycINFO, MEDLINE, ISI Web of Knowledge, CINAHL, and ProQuest Dissertations and Theses. Members of the study team and a reference librarian at San Diego State University developed the following Boolean search string: (“condom use skill*” OR “condom use step*” OR “condom use abilit*” OR “condom use criteri*” OR “condom skill*” OR “condom abilit*” OR “condom step*” OR “condom use failures”) AND (“self-efficacy” OR “self efficacy” OR confidence OR “self concept”) AND (adolescen* OR youth OR teen* OR college OR university OR “young adult” OR “high school” OR “high-school). A total of 654 articles were collected from this search conducted by the lead investigator. A PRISMA flowchart [58], which outlines the complete review process, is provided in Fig 1, and a PRISMA checklist (S1 File) was used to adhere to the PRISMA guidelines.

#### Duplicate articles

We compiled metadata from every article into Zotero citation management software [59] and sorted studies into folders based on the database from which they were mined. Using DOI and title metadata, 24 duplicate articles were identified and reconciled. After this de-duplication process, 630 unique studies remained to be included in the screening process.

#### Screening articles

Using a web-based machine learning program, Rayyan [60], the lead investigator screened titles and abstracts for mentions of both “self-efficacy” and “skills,” and studies meeting these inclusion criteria were retained for further screening. Titles and abstracts were further screened for inclusion to ensure they examined the correct age range as well as to ensure they were quantitative research designs. Thirty (N = 30) articles met the inclusion criteria and were reviewed in full.

In addition to the database search, relevant articles were compiled from the reference lists of studies selected for the full-text review. Since this review also included unpublished or non-peer-reviewed studies (e.g., dissertations), relevant investigators were contacted via email as well as through professional email lists to solicit any existing manuscripts or unpublished data analyses that met the inclusion criteria. In instances where it was difficult to determine whether a study should be included, additional team members discussed the study and arrived at a decision. Our final sample of studies consisted of 19 articles that met the criteria for inclusion in the review and were subjected to a full data extraction and analysis.

#### Data extraction & synthesis

Using Qualtrics online surveys, a template was developed for efficiently extracting relevant data from each article during a full-text review. The survey template was structured to collect numerous types and formats of data ranging from open-ended text to multiple choice response options. Flexible response options were included to ensure meaningful data were captured from studies that varied from our expected format. The following data were extracted from each of the included studies and assembled into a table for comparison: author last name, publication year, journal name, theories/frameworks that informed the study, study design, sample size, sample age range, sample race/ethnicity, sample genders, sample sexual orientation, study location, type of skills assessment, psychometrics reported (e.g., evidence of reliability), self-efficacy operationalization, type of self-efficacy assessment, instrument used, and study results. Assessing risk of bias in individual studies was limited to noting whether investigators provided raw data which could be used to calculate effect sizes, and, if they did not provide raw data, whether they provided an appropriate effect size.

#### Coding studies

The studies were all independently coded by three team members (JF and either MR or RH). Each study was coded for study characteristics, such as sample and measurement instrument descriptors, as well as for effect size characteristics. Investigators were specifically interested in extracting reported bivariate associations between condom application skills and self-efficacy (e.g., Pearson’s correlations). Discrepancies between the coders were resolved during a reconciliation facilitated by a third, unbiased moderator (AK) resulting in a final agreement of 100%. Using R [61], additional agreement statistics were calculated using the irr package [62] for each coded variable across the included studies (κ = 1.00; ICC = 1.00). Both direct observation of condom application skills as well as proxy measures that estimated a person’s actual skills through knowledge of correct and incorrect steps were included in this review. We also classified any measurement instrument that assesses perceived confidence or ability to apply a condom as a measurement of self-efficacy.

#### Risk of bias and quality assessment

The quality of studies included in the systematic review was assessed by two independent raters (JF and AJ) using the revised Cochrane risk of bias tool (ROB-2) for randomized trials [63] and the AXIS tool for cross-sectional studies [64]. The ROB-2 was used for studies meeting the criteria for a randomized trial whereas the AXIS tool was used for the remaining non-randomized studies. The raters reconciled discrepancies through discussion resulting in 100% agreement in quality assessments.

#### Data synthesis

We compiled descriptive study characteristics from each article into a narrative review to highlight common and disparate features of each study. Additionally, characteristics of each included article are displayed in Tables 1 and 2.

**Table 1:** General characteristics of studies included in a systematic review assessing condom application skills and self-efficacy in youth.

Characteristic	n (%)
Age of Sample	
13–18 years	16 (84.2)
18–25 years	3 (15.8)
Gender Identity	
Only men/boys	1 (5.3)
Only women/girls	11 (57.9)
Both men/boys and women/girls	7 (36.8)
Racial/Ethnic Identity	
Asian	1 (5.3)
Black or African-American	13 (68.4)
Hispanic or Latino	1 (5.3)
White	3 (15.8)
Multiple races/ethnicities	1 (5.3)
Location	
United States	9 (47.4)
Bahamas	8 (42.1)
United Kingdom	1 (5.3)
India	1 (5.3)
Study Design	
Cross-sectional	8 (42.1)
Randomized controlled trial	11 (57.9)
Condom Application Skills Measurement	
Assessed using only a direct observation instrument	10 (52.6)
Assessed using only a proxy instrument	8 (42.1)
Assessed using both direct observation and a proxy instrument	1 (5.3)
Self-Efficacy Operationalization	
Assessed condom application self-efficacy	4 (21.1)
Assessed general condom self-efficacy	7 (36.8)
Not stated or unclear	8 (42.1)

**Table 2:** Detailed summary of included studies.

Authors, location, design	Journal	Sample size, participants	Skills Measurement Instrument	Number of steps/skills assessed	Self-efficacy Measurement Instrument	Intervention/control conditions	Reported association between skills & self-efficacy
Forsyth* [73], US, Cross-sectional	Health Psychology	Total (N) = 43 Undergraduate men 79% Caucasian 89% sexually active Mean age = 18.9 years old	Unspecified direct observation instrument	15	CUSES	-	r = 0.14
Murphy [74], US, Cross-sectional	Journal of Adolescence	Total (N) = 132 Heterosexual Sexually active men/boys and women/girls 14–21 years old	Unspecified direct observation instrument	8	Unspecified self-efficacy instrument	-	-
Crosby [13], US, Cross-sectional	Journal of Adolescent Health	Total (N) = 522 Sexually active African-American women/girls 14–18 years old	Unspecified direct observation instrument	6	Unspecified self-efficacy instrument	-	-
Lindemann* [31], US, Cross-sectional	AIDS and Behavior	Total (N) = 178 Undergraduates 106 women 71 men 18–23 years old 79% White	MOCUS	7	CUSES	-	r = 0.31 (women) r = 0.26 (men)
Lucenko [75], US, Cross-sectional	Journal of Child & Adolescent Substance Abuse	Total (N) = 363 (n) = 256 men/boys (n) = 103 women/girls juvenile offenders 13–18 years old 30.9% African-American, 9.5% White, 31.2% Hispanic, 9.2% Haitian, 19.2% Other ethnic backgrounds	Unspecified direct observation instrument	9	Unspecified self-efficacy instrument	-	-
Lang [76], US, RCT	Prevention Science	Total (N) = 522 Sexually active African-American women/girls 14–18 years old	Unspecified direct observation instrument	-	Unspecified self-efficacy instrument	HIV intervention (n = 251) General health promotion (n = 271)	-
Malow* [77], US, RCT	Journal of the Association of Nurses in AIDS Care	Total (N) = 246 (Baseline) (N) = 203 (follow-up) Haitian adolescents 13–18 years old 70% women/girls	Unspecified direct observation instrument	9	ARMS	General health education (n = 101) HIV education intervention (n = 145)	r = 0.24
Chen [78], Bahamas, RCT	International Journal of STD and AIDS	Total (N) = 1360 (Baseline) 1108 (Follow-up) Bahamian youth 10–13 years old (Baseline) 13–16 years old (Follow-up)	CUSC	7	Unspecified self-efficacy instrument	Intervention for parents & youth (n = 436) Intervention for youth, control for parents (n = 427) Control for youth and parents (n = 497)	r = 0.27
Sales [79], US, RCT	Journal of Women’s Health	Total (N) = 245 Sexually active African-American women/girls 14–18 years old Reporting depressive symptoms	Unspecified direct observation instrument	6	Unspecified self-efficacy instrument	HIV prevention condition (n = 126) General health promotion condition (n = 119)	-
Sarafian* [80], Bangladesh, India, Cross-sectional	Dissertation	Total (N) = 263 Women sex workers Mean age = 18.92 years	Unspecified direct observation instrument	4	CUSES		r = 0.15
Stanton [81], Bahamas, RCT	Journal of Adolescent Health	Total (N) = 1997 Bahamian youth 13–16 years old	CUSC	7	Unspecified self-efficacy instrument	Received grade 6 intervention and were part of 1^st^ longitudinal study (n = 379) Received grade 6 intervention and were not part of 1^st^ longitudinal study (n = 159) Received grade 6 control and were part of 1^st^ longitudinal study (n = 230) Received grade 6 control and were not part of 1^st^ longitudinal study (n = 82) Did not receive grade 6 intervention or control, and were not part of 1^st^ longitudinal study (n = 1147)	-
Wang [82], Bahamas, RCT	AIDS and Behavior	Total (N) = 1360 (Baseline) 1115 (Follow-up) Bahamian youth 10–13 years old (Baseline) 13–16 years old (Follow-up) 53% women/girls	CUSC	8	Unspecified self-efficacy instrument	HIV intervention condition (n = 863; baseline) Control condition (n = 497; baseline)	χ^2^ = 11.77
DiClemente [83], US, RCT	Women and Health	Total (N) = 188 Sexually active African-American women/girls 13–17 years old Juvenile detention center	Unspecified direct observation instrument	7	Unspecified self-efficacy instrument	HIV risk reduction intervention (n = 95) Usual care control condition (n = 93)	-
Wang [84], Bahamas, RCT	AIDS Education and Prevention	Total (N) = 2564 Bahamian youth 13–16 years old (Baseline) 14–17 years old (Follow-up)	CUSC	8	Unspecified self-efficacy instrument	Both youth and parents received sexual health intervention (n = 664) Youth received sexual health intervention; parents received control condition (n = 559) Youth received sexual health intervention; parents did not receive any intervention (n = 569) Youth received standard of care; parents received no intervention (n = 772)	-
Hurrell [85], UK, Cross-sectional	Dissertation	Total (N) = 31 Undergraduate women Psychology students 18.5–21.5 years old	MOCUS, CUSC	7, 8	CUSES	Rehearsal session (n = 4) Information-only condition (n = 9) Skills-based condition (n = 8) Control condition (n = 7) Void control condition (n = 3)	ρ = 0.324 (CUSC & CUSES) ρ = 0.248 (MOCUS & CUSES)
Stanton [86], Bahamas, RCT	American Journal of Public Health	Total (N) = 2564 Bahamian youth 13–16 years old (Baseline) 14–17 years old (Follow-up)	CUSC	8	Unspecified self-efficacy instrument	Both youth and parents received sexual health intervention (n = 664) Youth received sexual health intervention; parents received control condition (n = 559) Youth received sexual health intervention; parents did not receive any intervention (n = 569) Youth received standard of care; parents received no intervention (n = 772)	-
Stanton [87], Bahamas, RCT	AIDS and Behavior	Total (N) = 2564 Bahamian youth 13–16 years old (Baseline) 14–17 years old (Follow-up)	CUSC	8	Unspecified self-efficacy instrument	Both youth and parents received sexual health intervention (n = 664) Youth received sexual health intervention; parents received control condition (n = 559) Youth received sexual health intervention; parents did not receive any intervention (n = 569) Youth received standard of care; parents received no intervention (n = 772)	-
Wang [88], Bahamas, Cross-sectional	Implementation Science	Total (N) = 4411 (Baseline) 4168 (Follow-up 1) 3439 (Follow-up 2) 3256 (Follow-up 3) 10–13 years old Approx. half were women/girls Bahamian youth	CUSC	6	Unspecified self-efficacy instrument	Participants were both students and teachers in schools implementing an evidence-based intervention. No control group was used as the primary research question was assessing implementation characteristics and their effects on condom use behaviors.	r = 0.35 (Follow-up 1) r = 0.15 (Follow-up 2) r = 0.41 (Follow-up 3)
Yu [89], Bahamas, RCT	Journal of Adolescence	Total (N) = 1970 16–19 years old 40.61% were men/boys Bahamian youth	CUSC	8	Unspecified self-efficacy instrument	Participants were students who had previously received an evidence-based intervention. No control group was used as the primary research question involved evaluating a condom use mechanism model.	-

* Included in meta-analysis; CUSC = Condom Use Skills Checklist; MOCUS = Measure of Observed Condom Use Skills; CUSES = Condom Use Self-Efficacy Scale.

### Meta-analysis

Following data extraction, several studies were excluded from the quantitative analyses due to lack of appropriate effect size statistics, resulting in five studies included in the meta-analysis. Analyses were conducted using SPSS 24.0 [65] as well as macros developed for meta-analyses by Wilson [66]. To determine whether there was significant heterogeneity between the included studies, a Cochran’s Q statistic [67] and an I^2^ statistic [68] were calculated. Due to the low statistical power of the Q-test for rejecting homogeneity with few effect sizes from small samples, we attempted to fit both a fixed- and random-effects model to these data [68, 69]. The random effects model was appropriate for this examination since different populations or operationalizations of the outcome variables may have existed between studies, introducing additional, uncontrollable variance that may be difficult to detect using the Q and I^2^ statistic [69]. Correlation coefficients were transformed using Fisher’s *z* transformation. While it is common to assess the likelihood of various publication biases using a statistical test of asymmetry, the overall small sample that we used appeared too under-powered to provide a meaningful assessment [70–72].

## Results

### Systematic literature review

#### Study characteristics

A summary of characteristics of the 19 articles included in the systematic review is provided in Table 1. Studies included appeared in a variety of journals such as *AIDS and Behavior* (3; 15.7%) and *The Journal of Adolescent Health* (2; 10.5%). Two studies were conducted as theses in partial fulfilment of doctoral degrees and were not published in academic journals. In total, 4 of the 19 studies (21.1%) were not guided by any specific theory, whereas 15 studies (78.9%) reported at least 1 guiding theory. The most frequently cited theoretical/behavioral frameworks were the Protection Motivation Theory (8 studies; 42.1%) and Social Cognitive Theory (6 studies; 31.6%) Table 2 contains more detailed information about the 19 included studies.

#### Risk of bias and quality assessments

**Fig 2:**
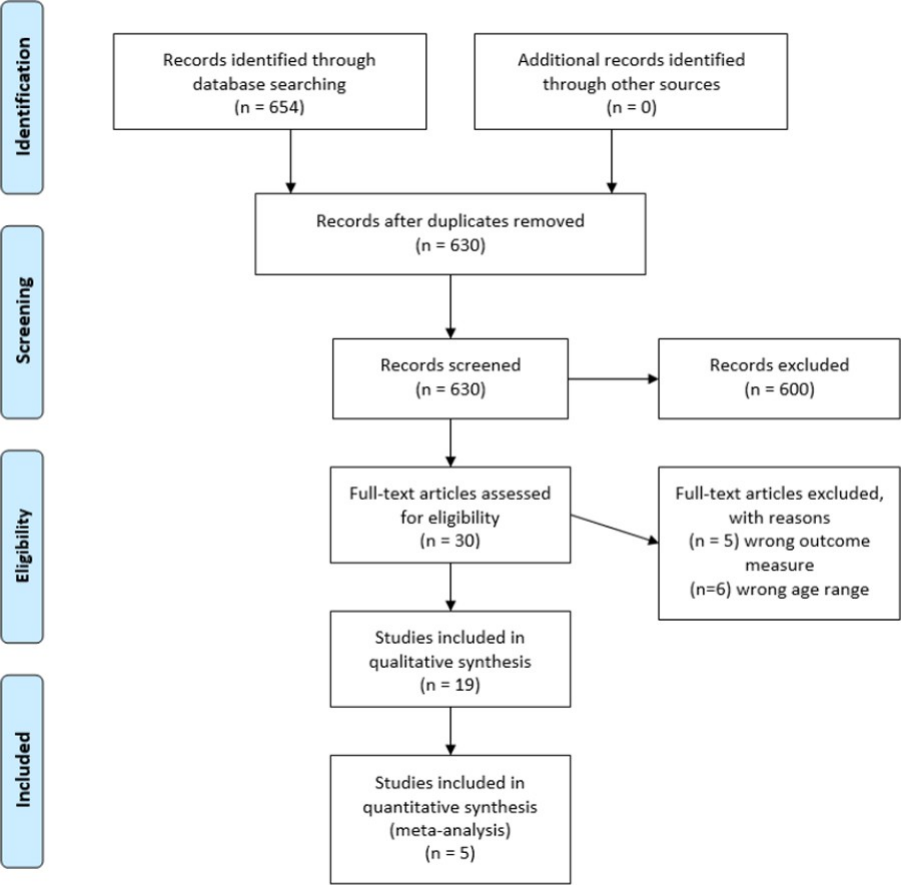
Risk of bias assessments of randomized trials included in the review.

Fig 2 depicts the results from the ROB-2 on randomized trials, which suggests that most randomized trials have some concerns that should be considered when making inferences based on their conclusions. Specifically, the randomization processes and the reported results in several studies were not presented effectively and lower the overall quality of the study and subsequent conclusions.

**Fig 3:**
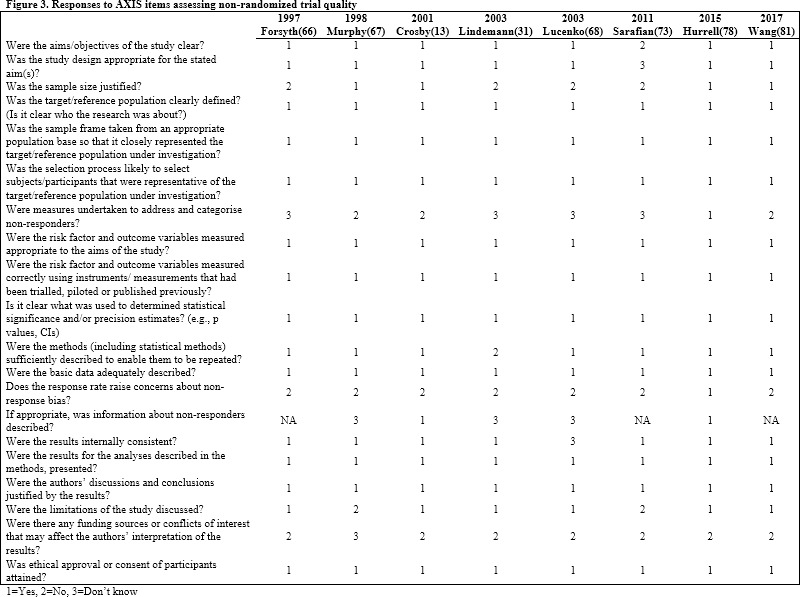
Responses to AXIS items assessing non-randomized study quality.

Fig 3 depicts the frequency of different responses to items on the AXIS quality assessment tool across all non-randomized trials included in the systematic review.

#### Condom application skills

More than half of the studies in this review (11 out of 19, 57.9%) assessed condom application skills through direct observation of the condom application. It should be noted that all direct observation instruments were completed by a study investigator instead of self-reported by the study participant. One study used both a direct observation instrument and a proxy instrument to assess condom application skills. Two studies out of those 11 directly assessing skills employed an existing instrument to directly assess skills. The remaining nine studies where skills were directly assessed used scales or checklists developed by the investigators on that research team. For studies directly assessing skills, the number of steps (for using a condom) assessed ranged from 4 steps to 15 steps, with most studies (six out of 11) citing between 7 and 9 steps. Only 3 out of the 11 studies utilizing a direct observation instrument (27.2%) reported reliability characteristics for the direct observation instrument employed (α = .78 to .93). All the studies that utilized a proxy instrument (9 out of 11, 81.8%) used one of 2 versions of the CUSC. Either 7 or 8 steps were considered crucial to correct condom application depending on which version was used. Out of the nine studies that used the CUSC, just over half (five out of nine; 55.6%) reported reliability characteristics for the CUSC (α = .42 - .83). It should be noted that all proxy instruments were self-report measures that were completed by study participants instead of by the study investigators.

#### Self-efficacy

Overall, five out of 19 studies (26.3%) used an existing measure to assess condom use self-efficacy (e.g., CUSES), and the remaining 14 out of 19 studies (73.7%) employed measures that were developed by the research team for that specific examination. With regards to condom use self-efficacy, four out of 19 studies (21.1%) used specific items related to condom application whereas seven out of 19 studies (36.8%) operationalized the construct in a more cursory sense (i.e., a single item assessing their ability to apply a condom or consisting mostly of items not related to application). The remaining eight studies either did not state how they operationalized self-efficacy or the operationalization could not be determined. Additionally, most studies (17 out of 19; 84.2%) reported reliability characteristics for the self-efficacy measurement instrument employed (α = .69 to .94).

### Meta-analysis

#### Association between skills and self-efficacy

**Fig 4:**
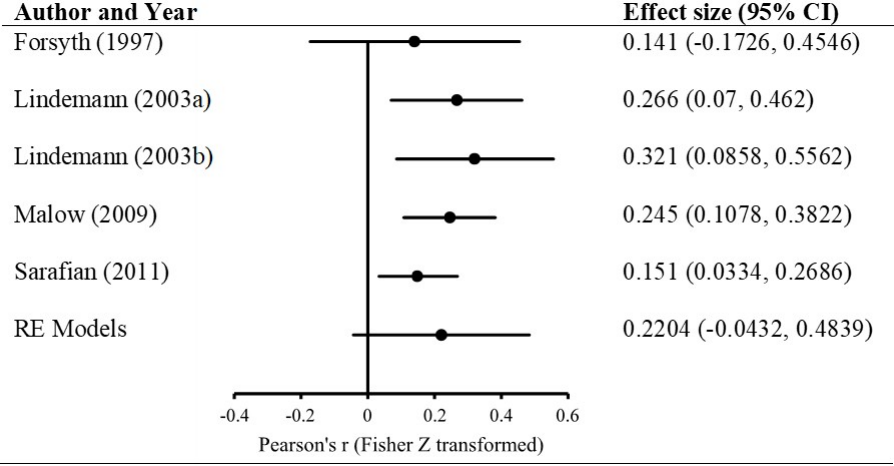
Forest plot of studies included in meta-analysis.

After coding the extracted data, 5 studies with a total sample size of 693 people contributed effect sizes for analyses of the association between condom application skills and self-efficacy. A random effects model was used to calculate an average effect size and 95% confidence interval. Results indicated a medium [90] but not statistically significant association between skills and self-efficacy, *r* = 0.217, 95% CI: -.043, .449, *z* = 1.639, *p* = .101. The studies were not found to be heterogeneous (*Q* = .2321, *p* = .994; I^2^ = 0%). Fig 4 depicts the distribution of standardized effect sizes of each study included in the meta-analysis as well as the results from the random effects model.

## Discussion

The purpose of this study was to examine the association between condom application skills and condom use self-efficacy in youth aged 13–25 years reported in both published and unpublished studies. The results from a random effects model indicated no statistically significant association (*r* = 0.217, *p* = .101) between skills and self-efficacy. To our knowledge, this is the first attempt to summarize existing data on the association between condom application skills and self-efficacy, and our results suggest that a person’s perceived confidence in their ability to apply a condom may not be as strongly associated with their actual skills as was previously thought.

One of the most enduring aspects of sexual health interventions is condom use, but upon which facets of condom use to focus remains challenging. While the current literature reports that interventions including a condom skills training component are effective, few studies report an outcome involving a skills assessment, opting instead to report consistency of condom use over time [34, 91, 92]. This may be due in part to policies that inhibit the implementation of condom application skill trainings and assessments in certain settings such as schools [33] suggesting that additional effort should be directed towards legislation that is informed by and supportive of scientific inquiry in this domain. Since condom application skills assessments can be challenging to administer, researchers develop and utilize more convenient measures targeting condom use self-efficacy which are often used in place of actual skills assessments. This practice assumes that these two concepts are interchangeable; however, our findings suggest that this is not empirically supported.

Regarding the theoretical impacts, Bandura linked these two constructs in his Social Cognitive Theory, which frequently has been used as a guiding theory in many public health and behavior change interventions [93–95]. Our results suggest that this linkage is not present in the context of condom application, and our reported effect size suggests that they are not as interchangeable as previously assumed. The absence of this association in our meta-analyses may suggest that the instruments included in the study examine two distinct constructs that are not well-aligned, possibly due to varying operationalizations of the constructs or selective reporting practices. This implies a more complementary nature between these two constructs instead of one of substitution for convenience as is often the case.

### Limitations

We recommend that additional research be undertaken to further examine the association between condom application skills and self-efficacy, as there were several limitations of the current study. The number of studies that fit the inclusion criteria for the meta-analysis was limited for several reasons. Many studies did not report effect sizes that could be used to calculate an association between condom use skills and self-efficacy. In these instances, our team attempted to contact the study investigators to inquire about these supplementary data, but we were overall unsuccessful in procuring them for several reasons (e.g., no response or unable to conduct analyses at that time). There were several experimental studies that reported appropriate effect sizes, but that also utilized multiple treatment or control conditions. This analysis was primarily concerned with the reported association between two measurement instruments instead of the overall effect of the intervention in different participant groups. Additionally, any study that involved assignment to one or more interventions would report the comparison to the same control group participants for each intervention. A similar issue arose with data from participants who were outside of our age range at baseline assessment which necessitated including data from only those timepoints in which they were aged 13–25 years. Even more studies may have been included had we not limited the inclusion criteria to English language articles which limited the number of studies in the review and analysis. This study also only assessed external condom application skills and self-efficacy which resulted in exclusion of any studies that focused on internal condom use. As a result of these characteristics, these studies were excluded from the meta-analysis, thus reducing our power to more robustly assess the association between our outcomes of interest and raising the probability that a Type-II error was committed. Due to the low-power of this meta-analysis, it was not prudent to conduct any moderation analyses (i.e., small between-study variability among so few studies severely limited the detection of heterogeneity; [96]).

Additionally, measurement instruments used to assess skills and self-efficacy were frequently developed by the investigators for that specific study, and these new instruments were not always well-defined (i.e., item development process). This may have resulted in studies using an instrument developed to assess a construct that was conceptually different from that of others included in the analysis. This would further culminate in a comparison of idiosyncratic conceptualizations of the outcomes of interest, creating a space for competing interpretations of the associations between them. Of those studies included in the meta-analysis, 3 used an existing scale and 2 used an instrument developed by their research team to assess skills. While this variation in measurement instrument may have introduced additional error, our assessment of homogeneity indicated that the included studies were not significantly different with regard to our outcomes of interest. Additionally, studies used the same general methodology for assessing self-efficacy (self-report questionnaires) and skills (self-report knowledge test proxy or a checklist of skills). Therefore, it is unlikely that an idiosyncratic conceptualization of application skills and self-efficacy are disparate enough to raise alarm in this study.

The range of reported reliability estimates suggests that the scales/instruments used across the included studies vary somewhat. Studies employing direct observation instruments (n = 3) reported Cronbach’s alphas ranging from 0.78–0.93 for data collected via those instruments, whereas the Cronbach’s alphas of self-efficacy instruments (n = 17) ranged from 0.69–0.94, indicating that these instruments range in their levels of internal consistency from below the acceptable alpha of 0.70 to more acceptable values [97]. Most concerning was that, of the five studies using a proxy instrument (all of which were the CUSC) to assess condom application skills that reported reliability characteristics, Cronbach’s alphas ranged from 0.42–0.83. An instrument with low internal consistency reliability often suggests that responses to items on the scale are not strongly correlated and participants are responding inconsistently–a problematic result given that reliability is necessary to establish construct validity [98]. This could be alleviated by removing or re-wording certain items on these scales so that they provide additional information related to the construct of interest (condom application skills or self-efficacy) that is not redundant of items already included.

The amount of studies that assess both condom application skills and condom use self-efficacy was small due to a lack of publications focusing on these two constructs simultaneously. During the initial screening process, 95 articles were excluded from the systematic review because they examined only self-efficacy for condom use without an appropriate skills component. That translates to approximately 67% of the sample being excluded for this reason alone. This was especially concerning considering the importance of both constructs in one of the most frequently utilized theoretical frameworks for studies in this review: the social cognitive theory (SCT). The conception of the SCT [20] included the construct of “appropriate skills” (p. 194) as a component of behavior change, yet only 18 studies in this review were found to measure and report on skills as well as self-efficacy. It seems logical that any intervention or study involving self-efficacy must also include a skills assessment of the same behavior in order to maintain at least partial theoretical fidelity.

Finally, few randomized trials included in this review exhibited a low risk of bias with the majority exhibiting some concerning characteristics that may have influenced the investigators’ findings. Specifically, most randomized trials lacked sufficient information describing deviations from intended interventions. Similarly, how investigators chose which outcomes to report was not always clear suggesting potential reporting bias in their studies. The cross-sectional studies were generally good quality with few demonstrating any glaring issues with the exception of very few mentions of how non-responses were handled in these studies.

### Implications

Despite this study’s limitations, there are important implications that may be used to shape current practices. The lack of studies reporting eligible effect sizes was especially limiting in our efforts to assess an association and points to a larger concern regarding reporting practices. Both the American Psychological Association (APA) and the American Education Research Association (AERA) have provided effect size reporting guidelines [99–101]. Since then, there have been improvements in the quality of effect size reporting, and yet there are still discrepancies in best practices, much to the frustration of reviewers and meta-analysts [102–104]. Even with professional associations and journals disseminating similar protocols for effect size reporting, there is no “hard-and-fast” rule regarding which effect sizes should be reported. Lakens [105] suggested that research questions and designs are primary drivers behind which effect sizes are reported. This implies that any approach to synthesizing reported effect sizes is often dictated by the initial investigators, thus putting the onus on such scholars to report all potentially salient data well in advance of a meta-analytic approach. While this seems like a heavy mandate to place on researchers, Thompson [106] posited that adopting a meta-analytic viewpoint, as demonstrated by consistently reporting effect sizes, is a positive step that would benefit future syntheses.

Most importantly, the sheer lack of articles assessing both condom use skills and condom use self-efficacy should be regarded as a high-priority issue in sexual health promotion endeavors. In this systematic review, 78% of all of the excluded studies did not assess condom use skills (n = 96) whereas 33% of all of the excluded studies did not assess condom use self-efficacy (n = 41). Additionally, 65% of the excluded studies only examined condom use-self-efficacy and did not examine condom use skills (n = 80). Assuming, without evaluation, that a behavior is being correctly performed may prove detrimental especially if participants are exposed to a “quantity over quality” approach to condom use wherein higher frequency of condom use is deemed an adequate metric for safer sex. It is also inappropriate to assume that a concept as cerebral as self-efficacy is an acceptable proxy for one’s actual ability without the requisite associative assessments between the two constructs. Sexual health promotion interventions and studies that have been eager to simplify the assessment of condom use skills should view this study as an impetus to further sharpen such tools. When we detach the concept of self-efficacy from demonstrated skills by assessing one and not the other, we see a fragmented picture of one’s behavior. Instead, future efforts should be devoted to developing brief measurement instruments for both constructs such that they can be employed in tandem. Special attention should be paid to assessing instruments among groups of women, men, and both women and men, as well as among other priority populations, such as men who have sex with men (MSM).

With regards to condom application skills, future directions could involve the development or application of innovative technologies from other fields such as virtual reality (VR) and augmented reality (AR) devices. Both VR and AR have emerged as potential complements to existing smartphone applications and immersive video games in addressing both mental and physical health areas [107–109]. Specifically, studies of surgeons who practiced surgical techniques using VR-simulated environments showed improved surgical skills compared to those who did not practice using VR devices [110–113]. Since these technologies aim to create an immersive environment within which the participant behaves, they may allow us to better assess condom skills in a cognitive state that better resembles real-world situations and their accompanying physical and emotional arousals (i.e., "hot" cognitive states; [114, 115]. Moving towards this type of thinking has the potential to yield a more robust assessment of how skills and self-efficacy can be targeted to promote healthier, safer sexual behaviors.

## Supporting information

S1 File PRISMA checklist. List of PRISMA items and where to find them in-text.
(PDF)
